# Use of *α*-Lactalbumin and Caseinoglycomacropeptide as Biopeptide Precursors and as Functional Additives in Milk Beverages Fermented by *L. helveticus*

**DOI:** 10.1155/2021/8822161

**Published:** 2021-04-13

**Authors:** Katarzyna Skrzypczak, Emilia Fornal, Dorota Domagała, Waldemar Gustaw, Ewa Jabłońska-Ryś, Aneta Sławińska, Wojciech Radzki, Anna Kononiuk, Adam Waśko

**Affiliations:** ^1^Department of Plant Food Technology and Gastronomy, Faculty of Food Science and Biotechnology, University of Life Sciences in Lublin, 8 Skromna Street, 20-704 Lublin, Poland; ^2^Department of Pathophysiology, Medical University of Lublin, 8b Jaczewskiego Street, 20-090 Lublin, Poland; ^3^Department of Applied Mathematics and Computer Science, Faculty of Production Engineering, University of Life Sciences in Lublin, 28 Głęboka Street, 20-612 Lublin, Poland; ^4^Department of Meat Technology and Food Quality, Faculty of Food Science and Biotechnology, University of Life Sciences in Lublin, 8 Skromna Street, 20-704 Lublin, Poland; ^5^Department of Biotechnology, Microbiology and Human Nutrition, Faculty of Food Science and Biotechnology, University of Life Sciences in Lublin, 8 Skromna Street, 20-704 Lublin, Poland

## Abstract

The objective of this investigation was to verify whether biologically active peptides (BAPs) could be obtained from water solutions of *α*-lactalbumin (*α*-la) and caseinoglycomacropeptide (CGMP) through an application of the new *Lactobacillus helveticus* strains. Also, the aim of this research was to determine the influence of addition of the analyzed protein preparations to milk subjected to fermentation by tested bacterial strains on the physicochemical properties of obtained milk beverages. The results indicate that CGMP is a more preferable source for the production of BAPs by the test bacteria than *α*-la. The antihypertensive and ACE inhibitory effects were the most widespread bioactivities among the detected BAPs. *α*-la containing fermented milk beverages had higher values of springiness, gumminess, chewiness, and resilience than analogous products containing CGMP, while CGMP-supplemented fermented products exhibited higher values of the hardness parameter. The highest values of hardness (0.416 ± 0.05 N) were recorded for beverages fermented by DSMZ containing the addition of CGMP, while the lowest value of this parameter (0.186 ± 0.06 N) was noted for products containing *α*-la and fermented by B734. Moreover, CGMP-containing fermented products were characterized by a generally higher value of the proteolysis index (PI) than analogous variants containing *α*-la. The use of analyzed strains and the selected protein preparations has a positive effect on the texture of fermented milk beverages and might contribute to an increase in the health-promoting potential of such products.

## 1. Introduction

Not only are milk proteins appreciated for their good nutritional quality, but they are perceived also as a reach precursor for a wide range of *in vivo* biologically active peptides exhibiting antilipidaemic, antimicrobial, mineral binding, and anticancer properties [1–4]. These findings prompt approaches based on application of biologically active peptides (BAPs) as one of the potential therapeutic tools leading to a decrease to some of disease-related risk factors of noncommunicable civilization disorders [5–7].

It is worth to mention that the cow milk proteins are also one of the major food allergens; however, the activity of effective proteolytic system of lactic acid bacteria (involved in milk fermentation) allows to reduce the antigenicity of the proteins [8]. Moreover, the amino acid sequences of BAPs derived from milk and whey proteins are able to modulate key biomarkers associated with the various systems of a human's body [6, 9]. Also, it is indicated that BAPs (including biopeptides derived from milk and whey proteins) might be involved in specific interactions with cellular receptors (or pivotal metabolic enzymes) that affect the modulation of specific physiological responses and influence the enhancement of a desired health-promoting effects [6–10].

Currently, a number of desired biological effects have been attributed to various sequences of BAPs (also depending on the type of the food matrix constituting a source of proteins that are precursors for biopeptides) [10–12].

Active amino acid sequences of biopeptides can be released from the native protein structure by enzymatic hydrolysis (*in vitro* or *in vivo*) involving various proteolytic enzymes. These biochemical transformations occur, among others, during the process of milk fermentation involving lactic acid bacteria bioactivities [2, 4, 6, 7].

It is worth to emphasize that among all the lactic acid bacteria, *L. helveticus* is characterized by the strongest proteolytic properties and is particularly appreciated for its protease and peptidase activities towards milk proteins [13, 14]. The microorganisms possess a complex proteolytic system enabling the generation of a wide range of short peptides (with diverse amino acid sequences) exhibiting various physicochemical characteristics and different functional properties [2, 3, 7].

Although it has been proven that certain biopeptides have significant nutritional and functional importance for the regulation of certain physiological functions in the organism, they have not been fully described and widely discussed in terms of their production, diverse mechanisms of action, and the wide range of multibiological activities [2]. Thus, BAPs generated from milk are the subject of a considerable amount of research and are of great interest to increasingly aware groups of consumers. Bioactive compounds that derive naturally from food have become desirable intermediate components not only in various sectors of food industry or production of nutraceuticals [15, 16] but also in medical, pharmaceutical, and vaccination applications [17].

The beneficial properties of BAPs derived from milk products are perceived as significant factors influencing a decrease in the risk of development of some civilization diseases [18]. Moreover, the various beneficial effects of milk and whey-derived BAPs (in the function of human body systems) and their low toxicity have prompted the development of the concept of applying these health-promoting components as functional ingredients in foodstuffs. This has special importance for prevention and treatment of many illnesses like inflammatory or cancer diseases, where small molecules able to interact with numerous pharmacological targets are applied. Moreover, many synthetic medical substances exhibit low selectivity, high toxicity, and involvement in undesired side effects (e.g., formation of toxic components after metabolic processes or other conversions and crossing of the brain-blood barrier); thus, the concept of incorporation of biopeptides with determined anti-inflammatory and anticancer activity in the treatment process is being developed [19–21]. Also, biopeptides incorporated in food products are important constituents of personalized food that can be used in the prevention and therapy of many diet-related and civilization diseases [10, 11, 22]. Therefore, the main aim of this research was to verify whether strains of *Lactobacillus helveticus* (T80, T105, and B734) could be applied to obtain BAPs from water solutions of *α*-lactalbumin (*α*-la) and caseinoglycomacropeptide (CGMP). Moreover, the investigation were focused on determination of the potential biological activities of the identified short amino acid sequences as well as on analysis of the selected physicochemical properties of milk beverages obtained by the addition of the tested protein preparations to milk and fermented by the new strains of *L. helveticus.*

## 2. Material and Methods

### 2.1. Bacterial Strains and Culture Conditions

Three strains of *Lactobacillus helveticus* (T80, T105, and B734) were used in the study. Previously, the isolated microorganisms were subjected to biochemical, microbiological, and genetic analyses in order to verify the identification, establish the taxonomic affiliation, and characterize selected properties like proteolytic activity and distribution of cell envelope proteinase genes [23–26]. The strains have not been commercially applied yet. *L. helveticus* DSMZ 20075 (DSMZ, Germany) was used as a reference strain in the study. The strains were stored at −80°C in a 15% solution of glycerol stock. Before analysis, the strains were systematically transferred (2% *v*/*v*) into sterile Man-Rogosa-Sharpe (MRS) broth (BTL sp. z o.o. Zakład Enzymów i Peptonów, Poland) containing 0.05 g/100 mL of L-cysteine (the pH of broth was adjusted to 6.3). The strain cultures were carried out overnight (anaerobically) at 42°C [27].

### 2.2. Preparation of Inoculums of Individual L. helveticus Strains

The bacterial inoculums used in the study were prepared according to Beganović et al. [28] with modifications. Samples containing 100 mL of fresh and sterile medium (MRS broths) were inoculated by overnight cultures of the individual *L. helveticus* strains (previously cultured in MRS broth) to obtain an optical density of OD_550_ = 0.5 (6 × 10^8^ cfu·mL^−1^). Thereafter, the inoculated samples were incubated at 42°C until the exponential phase of growth reached a value of OD_550_ = 0.8. Then, the bacterial biomasses were harvested by centrifugation (8000 x g/4°C/10 min), and the pellets were washed tree times using a sterile saline solution. The pellets were finally resuspended (also in saline) in order to obtain suspensions of the tested strains at an equal density level of OD_550_ = 0.7 (9 × 10^8^ cfu·mL^−1^). All these procedures were performed in sterile conditions.

### 2.3. Hydrolysis of Protein Preparations

Water solutions of *α*-lactalbumin (Arla Food, Denmark) and caseinoglycomacropeptide (Arla Food, Denmark) were prepared as described previously [26]. In brief, samples of water solutions (1% *w*/*v*) of the analyzed protein preparations were pasteurized in a water bath (80°C/30 min) and after cooling up to 35°C were inoculated (1% *v*/*v*) with the bacterial strain cell suspensions prepared previously. The tested samples were incubated at 42°C for 24 h and then subjected to heat treatment (100°C/5 min) in a water bath. Thereafter, the analyzed material was collected, filtered using a sterile syringe filter (*Ø* = 0.45 *μ*m), frozen at -80°C, lyophilized (Labconco, Kansas City, USA), and subjected to further examinations.

### 2.4. Liquid Chromatography-High-Resolution Mass Spectrometry (LC-HRMS) and Peptide Sequencing

The analysis was performed using an Agilent HPLC chromatograph series 1290 coupled to Agilent 6550 iFunnel Q-TOF equipped with a Jest Stream Technology electrospray ion source. An Agilent HPLC 2.1 × 100 mm column packed with Zorbax 300 SB-C18 1.8 *μ*m sorbent was applied for separation of peptides. The mobile phase consisted of 0.1% formic acid in water (A) and 0.1% formic acid in acetonitrile (B). The column flow rate was 0.5 mL/min, and the linear gradient from 3 to 95% B in 14 min was used, which was followed by 95% B for 1 min and a 2 min postrun at 3% B. The injection volume was 5 *μ*L, and column temperature was maintained at 40°C. Ion source gas (N_2_) temperature was 225°C; gas flow rate 12 L/min; nebulizer pressure 50 psi; sheath gas temperature 275°C; sheath gas flow 12 L/min; and capillary voltage set at 3500 V. The nozzle voltage was set at 1000 V and the fragmentor voltage at 400 V. Positive ions were generated and registered at the *m*/*z* range of 100–1700. The mass spectrometer operated at the dynamic extended the range mode. It was tuned using an Agilent ESI-L Low Concentration Tuning Mix (G1969-85000). Internal mass calibration was enabled by using the reference ions *m*/*z* 121.0509 and *m*/*z* 922.0098. Data were acquired in using Agilent Mass Hunter acquisition B.07.01 software while Agilent Mass Hunter qualitative analysis B.07 with integrated Bioconfirm add-on software and Spectrum Mill were implemented for data analysis and peptide mapping. Data were searched against Bovine milk NCBI protein database. Proteins and peptides were identified using nonspecific digestion, and a tolerance on the precursor and product mass measurements was set to 5 ppm and 50 ppm, respectively. A spectral quality filter was used with a precursor isolation purity of >70%. Search results were validated using the Auto Thresholds Strategy and Peptide Mode. The target FDR was set to 1.2%, which results in an actual FDR of 1%.

### 2.5. Determination of the Biological Activities of Detected Biopeptide Sequences

From the wide range of amino acid sequences obtained after hydrolysis of the analyzed protein preparations, products in the mass range from 400 to 8000 Da were selected for further *in silico* testing. The profiles of potential biological activities of the detected amino acid sequences were performed in accordance with the procedure included in the BIOPEP database [29–32] and BioPepDB [17, 33, 34]. Each of the identified peptides (with molecular mass in the investigated in the study range) was individually analyzed (among others with regard to sequence and molecular mass and also monoisotopic mass as well as type of peptide precursor) and was compared with the biopeptide sequences included in the databases together with their assigned and described bioactivities.

### 2.6. Fermentation of Milk with Addition of Protein Preparations and Texture Profile Analysis of Obtained Products

Samples of 13% regenerated skim milk (OSM Krasnystaw, Poland) enriched by the addition of 1% (*w*/*v*) of CGMP or *α*-la were used as a raw material submitted to fermentation by the tested strains of *L. helveticus.* The prepared variants of milk samples were pasteurized (80°C/30 min) in a water bath, cooled down to 37°C, inoculated (1% *v*/*v*) with a proper bacterial cell suspension (the inoculum of each strain was prepared according to the method described above), and transferred into tightly sealed sterile packages (in equal volumes of 40 mL). The process of fermentation was performed with the thermostatic method (42°C/12 h). Thereafter, the samples were cooled down to 4°C and maintained at this temperature for another 12 h before further analysis (texture profile analysis, assay of proteolysis index, determination of protein, and nitrogen content).

The texture profile analysis (TPA) was performed in order to compare the textural properties of final products (fermented beverages containing the addition of CGMP or *α*-la) obtained with the use of different protein preparations as well as various strains of *L. helveticus.* The hardness, springiness, cohesiveness, gumminess, and chewiness parameters were investigated with the use of TA-XT2i (Stable Micro Systems, Godalming, UK) according to the method described by Gustaw et al. [34]. The analyses were performed in triple repetitions.

### 2.7. Determination of the Protein Content, Nitrogen Content, and Assay of the Proteolysis Index

The protein content in the fermented products obtained was determined with the Kjeldahl method according to EN ISO 8968-1:2014 [35]. Determination of the non-protein nitrogen content was performed in accordance with ISO 8968-4:2001 [36], while the proteolysis index was calculated as follows: 100 × non‐protein nitrogen/total nitrogen [37]. Regenerated skim milk (RSM) without any addition and unfermented RSM containing 1% addition of CGMP or *α*-la were used as control samples. All analyses were performed in triple repetitions.

### 2.8. Statistical Analysis

Statistical analyses were performed using the Statistica 13.1 software package (StatSoft, Krakow, Poland). The hierarchical cluster analysis (HCA) was used to explore similarity between the examined samples. Clustering was performed using the Ward minimum variance method with Euclidean distance as a similarity measure. The principal component analysis (PCA) was applied to show the relationships between the variables. PCA was conducted using the correlation matrix. The results of PCA and the calculated Pearson correlation coefficients allowed choosing variables for HCA. Also, the post hoc test was applied in order to explore differences between the mean values in the analysis of texture parameters, determination of the protein, and nitrogen content and in the analysis of the proteolysis index (at 0.05 level).

## 3. Results and Discussion

### 3.1. Determination of the Biological Activities of Detected Biopeptide Sequences

Nowadays, an increasing interest of new food-derived sources of biopeptides precursors has been observed. Moreover, the development of interdisciplinary investigations focused on isolation and identification short amino acid sequences (containing from 2 to 20 residues) from various food matrixes in order to determine the peptide bioactivities is clearly discernible [38, 39]. This has contributed to the development of many bioinformatics tools that allow the detection and analysis of specific peptide sequences. Moreover, the investigations of determination of biologically active activities of peptides sequences might be performed in silico in accordance with the procedure provided in the created data bases that might be designed to analyze a wide range of biopeptides and their precursors or to determination-selected sources of proteins, e.g., milk bioactive peptide database [30–33, 40, 41]. The identification and analysis of peptide bioactivity presented in this article are also applied in the interdisciplinary research [30–33, 40–42]. The findings have revealed that diverse biopeptides can be also obtained through the hydrolysis of *α*-la and CGMP conducted with using selected *L. helveticus* strains (Table 1).

It is worth to mention that *L. helveticus* is the most proteolytic species among the genus of Lactobacillus and the most effective in producing vast multitude of BAPs exhibiting various beneficial therapeutic effects (including ability to reduce blood pressure as well as immunomodulatory, anti-inflammatory, antithrombotic, lipid-lowering, and anticholesterolemic properties) in the treatment of some diseases [2, 10–12, 43–46].

The analysis of obtained peptide sequences (generated by *L. helveticus* strains from the tested protein preparations) revealed the presence of a larger number of some amino acids, such as Pro (P), Glu (E), and Val (V), respectively, account for 18.20%, 8.38%, and 7.79%, whereas Met (M) and Asp (K) were the least abundant amino acids reaching 1.43% (each of them) of the number of total amino acids. The interaction between the amino acid composition of the peptides and antioxidant activity has been emphasized in the scientific literature. It was suggested that high proportion of hydrophobic amino acids influences higher antioxidant activity, compared to other hydrophilic amino acids. Moreover, research revealed the antioxidant properties of peptides might be reinforced through the presence of Trp, Tyr, and Pro [47]. This may suggest that detected antioxidative biopeptides VLPVPQK (Val-Leu-Pro-Val-Pro-Gln-Lys) and QKAVPYPQRDMPI (Gln-Lys-Ala-Val-Pro-Tyr-Pro-Gln-Arg-Asp-Met-Pro-Ile) seem to have a high potential to scavenging radicals (Table 1).

The findings demonstrated the presence of a wide range of BAPs with antihypertensive, ACE inhibitory, and antithrombotic activities in the fermented protein preparations. This is particularly relevant especially in the context of prevention and treatment of atherothrombotic disease, which affects a substantial part of society nowadays. It is considered that coronary artery disease and stroke are one of the world's leading causes of death. In our study, the antihypertensive effect was the most common bioactivity in all the detected sequences of BAPs (Table 2).

The desirable cardiovascular effects of BAPs derived from milk are associated with the presence of opioid peptides (generated from whey proteins *α*-lactalbumin and *β*-lactoglobulins), which influence blood pressure [48]. For example, *α*-lactorphin releasing from *α*-lactalbumin exhibits the potent activity lowering blood pressure in spontaneously hypertensive rats (SHR). Moreover, some BAPs (as caseinophosphopeptides) influence the reinforcement the solubility and absorption of calcium or exhibit antithrombotic effects through, e.g., inhibition the aggregation of ADP-activated platelets [49].

ACE (peptidyldipeptide hydrolase, EC 3.4.15.1) is an exopeptidase that performs an important role in the blood pressure regulation through the renin-angiotensin and bradykinin pathways [50]. ACE catalyzes the conversion of the angiotensin I to angiotensin II (an active vasoconstrictor octapeptide) that is able to interact with two receptors resulting in inducing the contraction of blood vessels that influence to blood pressure increase [51]. Therefore, inhibition of ACE contributed to an antihypertensive effect.

It was revealed that AVPYPQR (casokinin) obtained through enzymatic hydrolysis (by trypsin) of *β*-casein exhibited ACE-inhibitory and antihypertensive activity in SHR [52, 53]. It was also noted that the peptide concentration allowing inhibition of 50% of ACE activity was relatively low (IC50 = 15 *μ*M). Moreover, application of this BAP at the dose of 100 mg/kg induced reduction of systolic blood pressure (SBP) by -10 mm Hg in spontaneously hypertensive rats (SHR) [52, 54–56]. In addition, it is suggested that the sequence possesses antioxidative [57, 58] as well as immuno- and cytomodulatory activities [59]. This multifunctional biopeptide was present in the CGMP samples fermented by the strain T105 (Tables 1 and 2). Interestingly, it was the only *L. helveticus* strain able to release this BAP.

The YQKFPQY sequence is an example of another unique biopeptide that was obtained only through application of the particular *L. helveticus* strain. The sequence was detected only in the *α*-la samples hydrolyzed by T105. It has been confirmed that this BAP (released from milk protein through trypsin hydrolysis) exhibited SBP reducing effects (-15 mm Hg) in SHR when administered at the dose of 100 mg/kg [55, 60]. The present results have revealed that *L. helveticus* B734 was the only strain capable of generation of PYVRYL. The bioactive sequence was obtained from fermented CGMP (Table 1, column nos. 1 and 3). It is worth mentioning that this BAP exhibited an ability to decrease SBP by 23.4 mm Hg in SHR when applied at a dose of 3 mg/kg, which indicates its high therapeutic potential [60].

Unlike the above-mentioned BAPs (generated from the specific protein preparation only by some strains of the tested bacteria), the sequence MAIPPKK was released by all of the analyzed strains from CGMP (Table 1, column nos. 1, 2, and 3). The results of an *in vivo* study revealed that this biopeptide (characterized by antihypertensive and antithrombotic activities) derived from milk proteins by enzymatic hydrolysis (using trypsin) at the dose of 10 mg/kg decreased SBP in SHR by -28.0 mm Hg [61].

It was noted that the CGMP fermentation by *L. helveticus* B734 or T105 as well as the reference strain (DSMZ) yielded sequences with antihypertensive and ACE inhibitory activities (RPKHPIKHQ) exhibiting high therapeutic potential. The bioactivity of this peptide has been proved in an *in vivo* study, where the BAP administration at a dose of 6.1 mg/kg reduced SBP (-9.3 mm Hg) in SHR [56, 62]. This biopeptide was also detected in the *α*-la samples hydrolyzed by B734, T105, and T80 (Table 1). Another study [63] indicated that this BAP was also generated from *α*_s1_-CN by ten strains of *Streptococcus thermophilus* as well as the sequence RPKHPIKHQGLPQEVLNENLLRF (exhibiting immunomodulating and antibacterial activity). However, only three out of ten *S. thermophilus* strains (LMD-9, PB385, and 4F44) demonstrated the ability to release RPKHPIKHQGLPQEVLNENLLRF from the structure of the milk protein [63]. Similarly, some differences in the ability to produce this sequence were noticed among the *L. helveticus* tested in our study. The BAP was present in CGMP hydrolyzed by *L. helveticus* T80 and in all samples of protein preparations fermented by strain B734 (Table 1).

The YQEPVLGPVRGPFPIIV is an example of a biopeptide that was generated by all the *L. helveticus* strains tested, regardless of the type of substrate used for hydrolysis. The presence of this immunomodulating and antibacterial biopeptide [64] has been confirmed in all samples of *β*-CN hydrolyzed by *S. thermophilus* strains tested by Miclo et al. [63]. Moreover, an *in vivo* study (with murine models) revealed that this BAP stimulated regulation of major histocompatibility complex (MHC) class II antigen expression on bone marrow-derived macrophages and enhanced their phagocytic activity (inducing low release of cytokines) [65]. This suggests that YQEPVLGPVRGPFPIIV influencing modulation of macrophage properties may exhibit anti-infectious immunostimulating activity without proinflammatory effects.

Another biopeptide (that is a precursor of YQEPVLGPVRGPFPIIV) with immunomodulatory properties was LLYQEPVLGPVRGPFPIIV detected in all samples of the tested preparation fermented by the analyzed *L. helveticus* strains (Table 1). This BAP was also present in fermented products analyzed by Miclo et al. [63], who attributed mitogenic activity to this sequence as well.

The result revealed that only strains T80 and DSMZ were able to generate the biopeptide with mineral-building activity (RELEELNVPGEIVESLSSSEESITR), which was released from CGMP (Tables 1 and 2). It has been indicated that this BAP exhibits high affinity toward iron atoms due to its sequence (it includes 4 phosphoserine residues), and the mineral-building properties of this phosphopeptide have already been confirmed in *in vivo* studies (in rats and humans) [66–68]. Moreover, investigations conducted in animal models revealed that RELEELNVPGEIVESLSSSEESITR increased the efficiency of absorption and bioavailability of iron. The first clinical study comparing Fe/*β*-CN f (1-25) and iron sulfate showed that, 7 days after application of the preparations, a higher quantity of iron stored in organs was noted in the group that received the Fe/*β* (1-25) complex [66].

The peptide MAIPPKKNQDK was the only sequence with antithrombotic activity detected in the present study. It was generated from CGMP by all the tested strains (Table 1, column nos. 1 and 3). It is worth mentioning that the efficacy of this BAP has been demonstrated in other studies, and it has been established that the biopeptide inhibited ADP-induced platelet aggregation and fibrinogen binding in a concentration-dependent manner [69].

The present results revealed the ability to release individual peptide sequences by *L. helveticus* is a strain-dependent feature that is also in accordance with Broadbent et al. [70] and Barker et al. [7]. This may be related to differences in the specificity and efficiency of the proteolytic system of individual bacteria strains, which is consistent with the findings reported by other authors [71], who analyzed the ability of thermophilic lactic acid bacteria (LAB) to hydrolyze *β*-lactoglobulin and *α*-lactalbumin (in a chemically defined medium). Their results indicated visible diversification in the patterns of profiles of peptides was generated by LAB. It was explained by differences in proteinase specificities exhibited by the bacterial strains. Furthermore, it was claimed that the extent of hydrolysis not only depends on the bacterial strain used (its proteolytic properties) but is also influenced by the duration of the fermentation process and the type of protein substrate [10, 11, 72].

In our study, CGMP proved to be a better source of precursors for these biologically active peptides than *α*-la (Table 2). The present results correspond with those reported by Miclo et al. [63], who showed that the number of generated peptides (after hydrolysis of *β*-, *α*_s1_-, and *α*_s2_-caseins) was markedly diverse among ten strains of *Streptococcus thermophilus* (only 38 peptides were liberated by strain 4F44, whereas 65 peptides were generated by strain LMD-9). Among the examined *L. helveticus*, the greatest number of BAPs (29 different amino acid sequences) was generated by strain T105 from CGMP as well as by strain B734 (also from the same precursor of BAP), while the lowest number of biopeptides (8) was obtained from *α*-la hydrolyzed by strain T80 (Table 2). Also, it is worth to mention the *L. helveticus* T105 and B734 and allowed to obtain (from tested protein preparations) the highest amount of BAPs (14 sequences for each of the strain) of lower molecular weight (ranging below 800 Da) that might be related with the high enzymatic activity of these microorganisms. Moreover, previously performed analyses of proteolytic activity of the tested strains and study on genetic determinants of tested bacteria ability to hydrolyze proteins exhibited that among the tested strain collection the T105 was distinguished by high values of enzymatic activity as well as the number and diversity of combination the genes coding cell envelope proteinases [24]. That may also affect the results obtained in the presented investigation including the profile of BAP sequences received by applying *L. helveticus* T105.

The results indicate that certain sequences were released from the specified protein preparations only by one (determined, single) strain of the tested *L. helveticus*. This suggests that, depending on the strain used, it is possible to obtain a fermented protein preparation with different functional properties (corresponding to the different types and properties of BAPs released by bacterial enzymes). This is consistent with findings described by Ahn et al. [72], who additionally noted a stronger ACE inhibitory ability of whey fermented by *Lactobacillus brevis* than those fermented by *L. acidophilus*, *L. bifermentan*, *L. casei*, *L. helveticus*, *L. lactis*, *L. paracasei*, *L. plantarum*, and *L. reuteri.*

The obtained findings correspond to other study indicating that in *L. helveticus* the distribution of cell envelope proteinases, proteolytic activity, and ability to generate BAPs is strain-dependent [7, 70, 73, 74].

### 3.2. Comparison of the Texture Properties of Fermented Beverages

It was revealed that addition of selected milk or whey protein preparations (in a proper concentration) to milk may improve the texture parameters of fermented milk beverages and stimulate the growth of probiotic microorganisms [34, 71, 75].

In terms of textural properties, there were differences among the fermented milk beverages (Table 3). The products containing *α*-la fermented by strain B734 exhibited the highest value of such parameters as springiness, resilience, and cohesiveness, while the CGMP-containing products fermented by DSMZ were distinguished by the highest (Table 3).

**Figure 1:**
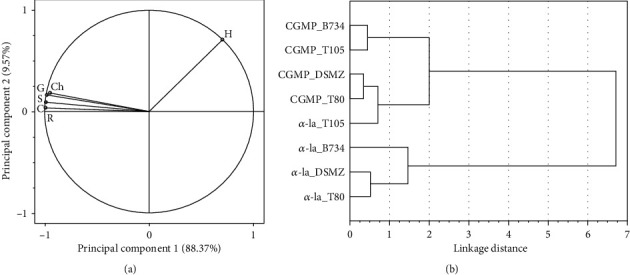
Projection of variables on the principal component plane ((a) H: hardness; Ch: chewiness; G: gumminess; S: springiness; C: cohesiveness; R: resilience) and dendrogram showing the results of hierarchical cluster analysis comparing the texture profiles of fermented milk beverages (b).

Considering that comparison of tested products in terms of all tested texture parameters simultaneously was also the interest of investigation, the HCA was applied to verify whether the groups of similar objects could be distinguished among all tested fermented products based on the analyzed texture features. Grouping the objects (fermented products) in terms of similarity of texture characteristics (Figure 1(b)) was carried out using the Ward method (applying a distance matrix formed based on the Euclidean distance). Since the analyzed features (texture parameters) were correlated (determined by analysis of the values of Pearson correlation coefficients), PCA was performed in order to present their structure (Figure 1(a)).

The results revealed that, apart from hardness, other texture parameters constituted one group of strongly positively correlated variables (the analysis performed in 3 repetitions for tested combinations). Hardness and springiness (which represented the remaining variables and most strongly correlated with them) were selected for cluster analysis.

The analysis indicated that, in terms of textural similarity, the fermented milk beverages could be assigned into two separate groups (bonding distance equal to 2.25). The first group includes milk beverages containing the addition of *α*-la and fermented by one of the strains: T80, DSMZ, or B734 (products obtained with T80 and DSMZ were the most similar to each other), whereas the other fermented milk beverages form a separate second group (Figure 1(b)).

All products classified in the first group were characterized by the highest values of the springiness parameter (in a range from 0.758 ± 0.602 for products containing *α*-la fermented by strain B734 to 0.477 ± 0.340 for beverages with *α*-la addition fermented by T80) and the lowest values of gumminess and chewiness (Table 3). In turn, the products from the second group exhibited the highest values of hardness. The highest values of this parameter (0.416 ± 0.054 N) were recorded for the beverages fermented by DSMZ containing addition of CGMP, while the lowest value (0.186 ± 0.063 N) was noted for the *α*-la-containing products fermented by B734, which belong to the first group (Table 3 and Figure 1(b)). Furthermore, the beverages grouped in the second cluster also demonstrated lower values of cohesiveness, springiness, gumminess, chewiness, and resilience (compared to products from the first group).

In general terms, the profile of mean values of the tested texture parameters exhibited by products fermented by T105 containing *α*-la (Figure 1(b)) demonstrated the same character as the profiles of the fermented products with CGMP addition (hardness is higher than springiness), while the rest variants of fermented beverages containing *α*-la exhibited a different shape of textural profile (the mean values of springiness are much higher than hardness). Moreover, it was noted that the fermented *α*-la-containing milk beverages exhibited higher values of springiness, gumminess, chewiness, and resilience, while the CGMP-containing products exhibited higher hardness values, which is in agreement with results described by Kozioł et al. [75]. They noted that supplementation of skim milk by 1% addition of CGMP yielded products with a higher value of the hardness parameter in comparison to 1% addition of *α*-la. On the other hand, it has also been noticed that 1% addition of *α*-la to whole milk may significantly shorten the time of milk coagulation (formation of acidic milk gel) during fermentation conducted with the use of *L. acidophilus* LA-5 (probiotic strain).

The differences observed in the texture characteristics between the fermented milk beverages containing *α*-la and CGMP might be related to the different levels of their hydrolysis conducted by the *L. helveticus* strains. The higher values of hardness exhibited by the product containing CGMP might be associated with a higher number of released peptides generated during the fermentation process. Moreover, the obtained results might be explained by Zhao et al. [76] indicating the molecular mass of the hydrolysates (formed during the fermentation from protein preparations) is related to the texture properties of products (positively influence on values of some of the textural parameters). It is worth to mention that more sequences of BAPs were detected in the CGMP samples hydrolyzed by the tested *L. helveticus* strains than in the *α*-la hydrolysates (Tables 1 and 2). These findings are probably associated with produced (during fermentation) peptides, which influence the structure of the gel network perceived as a dynamic system by establishment of various interactions between each other (and other compositions of the matrix including milk protein, denatured whey proteins, and calcium phosphate crosslinks) [77].

### 3.3. Determination of the Protein and Nitrogen Content and Comparison of the Proteolysis Index

The highest values of the proteolytic index were noted in samples of CGMP-containing milk beverages fermented by T105 and an analogous variant of milk products fermented by strain B734 (Table 4). Interestingly, the highest number of biopeptide sequences was detected in the CGMP samples hydrolyzed by *L. helveticus* T105 and in CGMP hydrolysates obtained using strain B734 (Tables 1 and 2).

In our study, this was noticed that fermented products with the addition of CGMP exhibited generally higher value of PI than the analogous variants containing *α*-la (Table 4), whereas hydrolysis of CGMP allowed to obtain more biopeptide sequences than *α*-la (Tables 1 and 2). The variations between the *α*-la- and CGMP-containing fermented products are probably induced by their different degrees of hydrolysis affected by differences in the substrate preferences (enzymatic specificity) of bacterial proteases. Moreover, the differences in hydrolysis intensity depending on the proteolytic activity of the applied strain of bacteria explain the differences in the number of formed BAPs and influence the content of non-protein nitrogen (through further enzymatic processes of conversion the peptide sequences into amino acids) [76].

**Figure 2:**
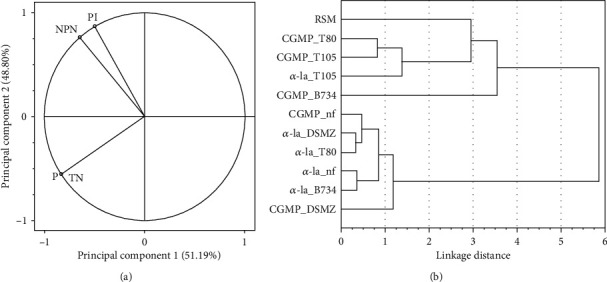
Projection of variables on the principal component plane (a) and dendrogram expressing the results of hierarchical cluster analyses of similarities of products in terms of the proteolysis index and contents of protein, total nitrogen, and non-protein nitrogen (b).

The HCA was used to separate samples exhibiting similar properties in terms of P, TN, NPN, and PI. The principal component analysis was used to illustrate the structure of the variables (Figure 2(a)).

A very strong correlation (determined by the analysis of the values of Pearson correlation coefficients) was noted between TN and P and between NPN and PI (the analysis was performed in 2 repetitions for the tested combinations). Therefore, the TN and PI (which was weaker correlated with TN than NPN) were selected for the cluster analysis.

Clustering was carried out with the Ward method using a Euclidean distance matrix and the analysis of the bond distances (2 bond level) revealed that 4 groups of similar products can be distinguished (Figure 2(b)). The first group included products containing addition of CGMP and fermented by strain DSMZ, products supplemented by *α*-la and fermented (separately) by strains B734, T80, and DSMZ and unfermented (control) CGMP and *α*-la samples.

However, the greatest similarity was observed between the *α*-la-supplemented products fermented by strain T80 (*α*-la_T80) and samples fermented by DSMZ (*α*-la_DSMZ). A close resemblance was also noted between unfermented *α*-la samples (*α*-la nf) and *α*-la-containing milk products fermented by strain B734 (*α* -la_B734).

The second group is represented only by CGMP-containing milk beverages fermented by *L. helveticus* B734 (Figure 2(b)). Furthermore, this variant of product was clearly different from the others and was characterized by the highest values of the PI, i.e., 29.53 ± 1.17% (Table 4).

The other fermented milk beverages constituted the third cluster (group III) and exhibited high values of PI as well (Table 4). Moreover, the greatest similarity was observed between CGMP-containing products fermented by strain T80 and an analogous milk variant fermented by *L. helveticus* T105 (Figure 2(b)). In turn, the unfermented RSM that was the most distinct from the other samples (in terms of the analyzed parameters) constituted a separate group (IV).

The differences between the fermented products observed in our study might be related to the different metabolic activities of the strains, their enzymatic preference for selected protein preparations influencing texture profiles, proteolysis index, and contents of protein, total nitrogen, and non-protein nitrogen. This is in agreement with the results reported by Ewe and Loo [78], who determined the effects of metabolic activities of *L. helveticus* on the texture of milk products and noted changes in crude macronutrients and the resultant modification of textural properties of cream.

## 4. Conclusions

The tested strains of *L. helveticus* exhibit a particular ability to generate a wide range of biopeptides with antihypertensive, ACE inhibitory, and antithrombotic activities during the hydrolysis of *α*-lactalbumin and caseinoglycomacropeptide. However, CGMP turned out to be preferred by the analyzed bacteria than *α*-la, as more sequences of BAPs were generated from CGMP. Moreover, the application of CGMP in production of milk beverages fermented by the tested strains can provide products with higher values of the hardness parameter and the proteolysis index in comparison to analogous products containing *α*-la.

The present obtained results suggest that the novel strains of *L. helveticus* strains (especially B734 and T105) might be applied as adjunct cultures in the production of fermented milk-derived products supplemented with selected milk or whey protein preparations and what may contribute to enhance the functional properties of such foodstuffs.

## Figures and Tables

**Table 1:** Sequences of biopeptides detected in the hydrolysates.

Source of peptides (type of analyzed hydrolysate)	*L. helveticus* strain	Identified peptide sequence	Mass (Da)	ID of the bioactive peptide in the database
CGMP	T105	LPYPYY	814.30	biopep00859/BioPepDB^b^
CGMP	T80	LRF^a^	434.26	biopep00880/BioPepDB
CGMP	T105	DKIHPF	755.39	biopep00136/BioPepDB 7493/BIOPEP-UWM^c^
CGMP	T105	AVPYPQR	829.44	biopep00091/BioPepDB biopep04785/BioPepDB biopep04775//BioPepDB 3480/BIOPEP-UWM 7875/BIOPEP-UWM
CGMP	DSMZ, T80, T105	DKIHP	608.33	biopep00135/BioPepDB
CGMP	T80	EMPFPKYPVEP	1332.64	biopep00178/BioPepDB
CGMP	B734	RPKHPI	746.45	biopep01209/BioPepDB
CGMP	B734	PYVRYL	809.44	biopep01134/BioPepDB
CGMP	B734	LTLTDVE	789.41	biopep00907/BioPepDB 8155/BIOPEP-UWM
CGMP	T80	KYPVEPFTESQSLTL	1737.88	biopep00721/BioPepDB
*α*-la	DSMZ			
CGMP	B734	GPFPIIV	741.44	8159/BIOPEP-UWM biopep00385/BioPepDB
CGMP	T105	FVAPFPEVFGK	1236.65	biopep00304/BioPepDB
CGMP	B734, T80	IPNPIGSE	825.42	biopep00561/BioPepDB
CGMP	B734, DSMZ, T105	ARHPHPHLSF	1197.61	biopep00073/BioPepDB
CGMP	B734, DSMZ, T80, T105	SPPEIN	655.30	biopep01254/BioPepDB
*α*-la	T105	YQKFPQY	972.47	biopep01624/BioPepDB 9254/BIOPEP-UWM
CGMP; *α*-la	B734, DSMZ, T80, T105	ERF^a^	450.22	biopep00189/BioPepDB
CGMP	B734, DSMZ, T80, T105	VRSP	457.26	biopep01460/BioPepDB 8309/BIOPEP-UWM
*α*-la	T80			
CGMP	T80, T105, B734,	SRY^a^	424.21	biopep01260/BioPepDB
*α*-la	T80, T105, B734, DSMZ			
CGMP; *α*-la	B734, DSMZ, T80, T105	RPKHPIKHQGLPQEVLNEN	2233.22	biopep01215/BioPepDB
*α*-la	B734	RPKHPIKH	1011.60	biopep01211/BioPepDB
CGMP	T105	RPKHPIKHQGLPQEV	1762.99	biopep01214/BioPepDB
CGMP	B734, DSMZ, T105	RPKHPIKHQGLPQ	1534.88	biopep01213/BioPepDB
CGMP	B734, DSMZ, T105	RPKHPIKHQ	1139.6	7483/BIOPEP-UWM biopep01212/BioPepDB
*α*-la	B734, T80, T105			
CGMP	B734, T80, DSMZ, T105	HKEMPFPKYPVEPF	1744.86	biopep00457/BioPepDB
CGMP	B734, T105	MAIPPK	655.37	biopep00945/BioPepDB 3293/BIOPEP-UWM
CGMP	B734, T80, DSMZ, T105	MAIPPKK	783.46	biopep00946/BioPepDB 3294/BIOPEP-UWM^c^
CGMP	B734, T80	IPNPIGSE	825.42	biopep00561/BioPepDB
CGMP	B734, T105	RELEE	674.32	biopep01181/BioPepDB
CGMP	T80, T105	QKAVPYPQRDMPI	1541.80	biopep01145/BioPepDB
CGMP; *α*-la	B734, T80, DSMZ, T105	AYPS	436.20	8472/BIOPEP-UWM 8380/BIOPEP-UWM
CGMP	B734, DSMZ	LLR^a^	400.28	8484/BIOPEP-UWM biopep00827/BioPepDB
CGMP	T80, DSMZ	RELEELNVPGEIVESLSSSEESITR	2801.39	biopep04772/BioPepDB 3055/BIOPEP-UWM
CGMP; *α*-la	B734, T80, DSMZ, T105	YQEPVLGPVRGPFPIIV	1880.06	biopep04801/BioPepDB biopep04091/BioPepDB biopep01621/BioPepDB
CGMP	B734, DSMZ T105	NLHLPLP	802.47	2669/BIOPEP-UWM biopep01010/BioPepDB
CGMP; *α*-la	B734, T80, DSMZ, T105	VTSTAV	576.30	7481/BIOPEP-UWM biopep01475/BioPepDB
CGMP; *α*-la	B734, T80, DSMZ, T105	LLYQEPVLGPVRGPFPIIV	2106.22	8174/BIOPEP-UWM
*α*-la	T105, DSMZ,	LLYQEP	761.40	biopep00829/BioPepDB
CGMP	DSMZ	YPFPGPI	789.41	biopep04798/BioPepDB 7665/BIOPEP-UWM 3262/BIOPEP-UWM
CGMP; *α*-la	B734, T80, DSMZ, T105	MAIPPKKNQDKTEIPTINTIASGEPTSTPTTEAVESTVATLEDSPEVIESPPEINTVQVTSTAV	6703.37	biopep03480/BioPepDB
CGMP; *α*-la	DSMZ	YYQQKP	825.40	8383/BIOPEP-UWM
CGMP	DSMZ	YIPIQY	795.42	biopep01570/BioPepDB 7630/BIOPEP-UWM
CGMP	B734, T80, DSMZ, T105	MAIPPKKNQDK	1268.69	biopep04784/BioPepDB 2796/BIOPEP-UWM 3292/BIOPEP-UWM
CGMP	T105	VLPVPQK	779.49	biopep01419/BioPepDB 7877/BIOPEP-UWM
CGMP	B734, T80, DSMZ, T105	VPSERYL	862.45	9250/BIOPEP-UWM biopep01442/BioPepDB
CGMP	T80	VVPP	410.25	biopep01483/BioPepDB 8308/BIOPEP-UWM
*α*-la	T105	TKKTKLTEEEKNRL	1716.97	biopep01292/BioPepDB
CGMP	B734, T80,			
CGMP	B734, T80, DSMZ, T105	VQVTSTAV	803.40	biopep01445/BioPepDB 8264/BIOPEP-UWM
CGMP	B734, T80	RPKHPIKHQGLPQEVLNENLLRF	2762.54	biopep03712/BioPepDB
*α*-la	B734			

^a^Based on accurate mass matching, <5 ppm; ^b^database: http://bis.zju.edu.cn/biopepdbr/ [33]; ^c^database: http://www.uwm.edu.pl/biochemia/index.php/pl/biopep [29].

**Table 2:** Number of detected biopeptide sequences (with identified bioactivity) generated from the tested protein preparations by the analyzed *L. helveticus* strains.

Bioactivity	Source of peptides (type of analyzed hydrolysate)	Number of bioactive peptide sequences generated by the tested *L. helveticus* strains			
		B734	DSMZ	T80	T105
Antihypertensive	CGMP	24	17	18	24
	*α*-la	7	7	7	9
ACE inhibitory	CGMP	8	9	5	8
	*α*-la	3	3	4	4
Antithrombotic	CGMP	3	2	2	3
	*α*-la	—	—	—	—
Antioxidative	CGMP	3	2	3	4
	*α*-la	1	1	1	1
Antimicrobial/antibacterial	CGMP	4	3	4	3
	*α*-la	3	2	2	2
Immunomodulating/immuno- and cytomodulatory peptides	CGMP	2	3	2	3
	*α*-la	2	2	2	2
Mineral binding	CGMP	—	1	1	—
	*α*-la	—	—	—	—
Opioid agonist	CGMP	—	1	—	—
	*α*-la	—	—	—	—
Total number of identified different biopeptide sequences	CGMP *α*-la	29 11	24 11	26 8	29 12

**Table 3:** Texture parameters for analyzed fermented products.

Analyzed fermented product	Texture parameter					
	Hardness (N)	Cohesiveness	Springiness	Gumminess (g)	Chewiness (g)	Resilience
CGMP_B734	0.318	0.038	0.050	0.012	0.001	0.019
	±0.033	±0.005	±0.012	±0.002	±0.000	±0.003
CGMP_DSMZ	0.416	0.028	0.037	0.012	0.001	0.014
	±0.054	±0.006	±0.009	±0.001	±0.000	±0.003
CGMP_T80	0.390	0.03	0.042	0.010	0.001	0.015
	±0.193	±0.020	±0.037	±0.002	±0.000	±0.011
CGMP_T105	0.282	0.050	0.067	0.014	0.001	0.026
	±0.040	±0.014	±0.031	±0.004	±0.001	±0.007
*α*-la_B734	0.186	0.223	0.758	0.035	0.034	0.131
	±0.063	±0.152	±0.602	±0.020	±0.029	±0.093
*α*-la_T105	0.356	0.051	0.072	0.018	0.001	0.026
	±0.01	±0.002	±0.007	±0.001	±0.000	±0.001
*α*-la_DSMZ	0.236	0.179	0.544	0.042	0.029	0.100
	±0.006	±0.098	±0.346	±0.023	±0.024	±0.058
*α*-la_T80	0.273	0.145	0.477	0.038	0.023	0.079
	±0.051	±0.078	±0.340	±0.020	±0.020	±0.044

Explanation notes: the analyzed fermented products are described by the designations: the name of the additive introduced in the milk (*α*-la_ refers to products containing addition of *α*-lactalbumin; CGMP_ refers to products containing addition of caseinoglycomacropeptide) followed by the name of the *L. helveticus* strain that was used in fermentation; the results are given as the mean values ± standard deviation (*x̅*±*s*/SD). The post hoc test did not reveal any statistical differences among analyzed fermented products in terms of the tested texture parameter (values in the same column) at 0.05 level.

**Table 4:** Proteolysis index, protein, non-protein nitrogen, and total nitrogen contents in the obtained milk products.

Analyzed product	TN (g/100 g)	P (g/100 g)	NPN (g/100 g)	PI (%)
RSM	0.81 ± 0.11	5.18 ± 0.73	0.14 ± 0.01^a^	17.04 ± 1.60^a^
CGMP_nf	0.87 ± 0.05	5.53 ± 0.29	0.17 ± 0.01^c,d^	19.81 ± 0.76^b,c^
CGMP_B734	0.88 ± 0.04	5.61 ± 0.27	0.26 ± 0.01^h^	29.53 ± 1.17^f^
CGMP_DSMZ	0.85 ± 0.01	5.40 ± 0.20	0.14 ± 0.00^a^	16.99 ± 1.01^a^
CGMP_T80	0.83 ± 0.01	5.26 ± 0.07	0.22 ± 0.01^f^	27.18 ± 0.42^e^
CGMP_T105	0.84 ± 0.01	5.36 ± 0.07	0.24 ± 0.01^g^	28.28 ± 0.37^e,f^
*α*-la_nf	0.87 ± 0.03	5.52 ± 0.21	0.15 ± 0.01^a^	16.79 ± 1.03^a^
*α*-la_B734	0.86 ± 0.02	5.50 ± 0.13	0.16 ± 0.01^b^	18.16 ± 0.35^a,b^
*α*-la_DSMZ	0.86 ± 0.04	5.49 ± 0.26	0.18 ± 0.00^d^	20.56 ± 0.77^c^
*α*-la_T80	0.86 ± 0.01	5.46 ± 0.07	0.17 ± 0.01^c^	19.44 ± 1.23^b,c^
*α*-la_T105	0.84 ± 0.01	5.38 ± 0.06	0.19 ± 0.00^e^	22.71 ± 0.59^d^

Explanation notes: P: protein; TN: total nitrogen; NPN: non-protein nitrogen; PI: proteolysis index; the analyzed fermented products are described by the designations: the name of the additive introduced in the milk (*α*-la_ refers to products containing addition of *α*-lactalbumin; CGMP_ refers to products containing addition of caseinoglycomacropeptide) followed by the name of the *L. helveticus* strain that was used in fermentation; the results are given as mean values ± standard deviation (*x̅*±*s*/SD). Different letters in the same column indicate statistical differences at 0.05 level.

## Data Availability

All the experimental data used to support the findings of this research are available from the corresponding author upon request.
